# WiPIHT: A WiFi-Based Position-Independent Passive Indoor Human Tracking System

**DOI:** 10.3390/s25133936

**Published:** 2025-06-24

**Authors:** Xu Xu, Xilong Che, Xianqiu Meng, Long Li, Ziqi Liu, Shuai Shao

**Affiliations:** School of Computer Science and Technology, Jilin University, Changchun 130012, China; chexilong@jlu.edu.cn (X.C.); mengxq23@mails.jlu.edu.cn (X.M.); lilong21@mails.jlu.edu.cn (L.L.); ziqi20@mails.jlu.edu.cn (Z.L.); shaoshuai22@mails.jlu.edu.cn (S.S.)

**Keywords:** WiFi sensing, Channel State Information (CSI), indoor movement tracking, position independence, signal processing

## Abstract

Unlike traditional vision-based camera tracking, human indoor localization and activity trajectory recognition also employ other methods such as infrared tracking, acoustic localization, and locators. These methods have significant environmental limitations or dependency on specialized equipment. Currently, WiFi-based human sensing is a novel and important method for human activity recognition. However, most WiFi-based activity recognition methods have limitations, such as using WiFi fingerprints to identify human activities. They either require extensive sample collection and training, are constrained by a fixed environmental layout, or rely on the precise positioning of transmitters (TXs) and receivers (RXs) within the space. If the positions are uncertain, or change, the sensing performance becomes unstable. To address the dependency of current WiFi indoor human activity trajectory reconstruction on the TX-RX position, we propose WiPIHT, a stable system for tracking indoor human activity trajectories using a small number of commercial WiFi devices. This system does not require additional hardware to be carried or locators to be attached, enabling passive, real-time, and accurate tracking and trajectory reconstruction of indoor human activities. WiPIHT is based on an innovative CSI channel analysis method, analyzing its autocorrelation function to extract location-independent real-time movement speed features of the human body. It also incorporates Fresnel zone and motion velocity direction decomposition to extract movement direction change patterns independent of the relative position between the TX-RX and the human body. By combining real-time speed and direction curve features, the system derives the shape of the human movement trajectory. Experiments demonstrate that, compared to existing methods, our system can accurately reconstruct activity trajectory shapes even without knowing the initial positions of the TX or the human body. Additionally, our system shows significant advantages in tracking accuracy, real-time performance, equipment, and cost.

## 1. Introduction

Indoor human localization and activity trajectory tracking play a crucial role in various fields, including criminal investigation, security, daily activity monitoring, elder care, and location-specific device triggering. Currently, methods for indoor human activity localization include a series of techniques involving cameras, acoustics [1,2,3,4,5], infrared detection [6], trackers, sensor localization, and mobile phone positioning [7,8]. However, due to factors such as privacy concerns, equipment requirements, and environmental limitations, these methods are not suitable for all situations. In recent years, with the increasing research in wireless sensing, there have been significant breakthroughs in the field of indoor localization and activity trajectory tracking. Researchers have utilized WiFi signals for sensing and localization, but many existing methods based on WiFi signal characteristics use CSI fingerprints [9,10,11,12,13,14,15,16,17,18] for location recognition. This approach is highly correlated with the specific positions and movement directions of individuals indoors, often requiring extensive training samples collected from different locations and directions. Only after intensive training can these methods achieve desirable results. Moreover, the trained models are typically tailored to specific training environments and are significantly affected by environmental layouts, resulting in decreased accuracy or complete failure in positions not included in the training set.

Nonetheless, past research has provided valuable insights into WiFi indoor localization and tracking. These studies have shown that, during WiFi signal transmission, Channel State Information (CSI) varies over time due to the impact of object movements within the WiFi signal coverage area. By conducting an in-depth analysis of CSI, it is possible to identify the relatively unique effects of different movements on signal transmission. Different positions, movement directions, and movement speeds exhibit relatively unique characteristics, presenting distinct temporal variations. Therefore, by analyzing the temporal variations in CSI, it is possible to reconstruct the indoor positions and movement trajectories of humans.

Currently, there are several methods that use WiFi CSI signal analysis for passive tracking through object reflection which do not require extensive training data collection and training, such as WiDar [19] and IndoTrack [20]. These methods do not require extensive training like CSI fingerprinting does, and they calculate real-time positions through techniques that assess parameters such as the Angle of Arrival (AoA), Time of Flight (ToF), and Doppler Frequency Shift (DFS) [21,22]. However, each has its own drawbacks: they may require specific equipment, be cumbersome to use, or fail to achieve the desired level of precision. Most importantly, they all rely on the relatively precise positioning of transceiver devices in space. Therefore, we propose the WiPIHT system. This system leverages common commercial WiFi devices and can achieve high-efficiency, low-cost real-time indoor human activity trajectory shape reconstruction without extensive complex deployment, training, or additional hardware. It does not rely on the precise positioning of TXs and RXs, and it is unaffected by various environmental parameters such as lighting, obstructions, and layout. WiPIHT demonstrates superior performance compared to mainstream approaches (e.g., RSSI fingerprinting), not only in Dynamic Time Warping (DTW) metrics but, more critically, by fundamentally eliminating the resource-intensive training phase. This exempts the system from laborious site surveys, significantly reducing pre-deployment overhead. Concurrently, it overcomes fingerprinting’s inherent constraint of environment-specific calibration and operates independently of initial position knowledge. This intrinsic position-agnostic property ensures enhanced robustness and deployment adaptability in practical settings. For real-time processing, WiPIHT surpasses fingerprinting by obviating the computationally expensive fingerprint database matching, thereby achieving significantly higher trajectory reconstruction efficiency.

However, to achieve passive indoor movement trajectory tracking and localization based on WiFi CSI variations, we still need to address a series of technical challenges. First, we need to extract data that can reflect movement trajectories from CSI, which contains complex noise. Second, it is necessary to perform location-independent trajectory reconstruction feature extraction. Finally, a model must be established to reconstruct the extracted features into real-time indoor movement trajectory shapes. WiPIHT offers tailored solutions to address these challenges. For CSI denoising and extraction, we employ noise elimination methods including amplitude denoising and phase denoising, combined with Principal Component Analysis (PCA) to correct and separate noise from the raw CSI data. By analyzing the autocorrelation function (ACF) [23,24] of the CSI, we determine the real-time movement speed curves that vary with human activity over time. Using the principles of the Fresnel zone model and CSI phase rotation, we extract real-time movement direction features from CSI that are independent of location. For determining the initial position of the human body, we use triangulation based on the Multiple Signal Classification (MUSIC) algorithm [25,26,27]. We then detect the start position of each continuous movement in time using a sliding window approach based on the real-time speed obtained from the ACF. Finally, we establish a model to reconstruct the real-time indoor movement trajectory shapes using the real-time speed and direction curves.

The main innovation of this work lies in utilizing the impact of moving objects on WiFi signal variations. It extracts real-time speed curves for human movement that are location-independent from the autocorrelation function of the CSI. It also extracts real-time direction features that are location-independent using the Fresnel zone model and CSI phase rotation. By combining these two features, it achieves high-precision motion trajectory reconstruction. This approach not only addresses the dependency on specialized equipment and the risk of privacy leaks in indoor trajectory tracking and localization but also overcomes the problems of excessive reliance on environmental layouts and the high training costs of other WiFi-based indoor positioning systems. It also solves the drawback of being unable to extract motion trajectory outlines when the positions of the TXs and RXs are unknown. Through its effectiveness and real-time capability, this method takes a significant step towards realizing wireless sensing-based passive indoor motion trajectory tracking in practical scenarios.

In summary, we make the following contributions:

- Propose a method to extract human motion trajectory features using CSI by performing amplitude and phase error correction and denoising on raw CSI data, making the CSI data more representative of human activities.
- Utilize only two non-parallel WiFi device links to ensure real-time reconstruction of motion trajectory shapes, regardless of the position, speed, and direction of human movement, even when the relative positions of the TXs and RXs to the human body are unknown. Calculations of initial position and initial motion angle provide absolute trajectory coordinates.
- Implement WiPIHT on regular commercial WiFi devices and conduct extensive experiments and evaluations, validating the efficiency and high precision of the trajectory reconstruction. Evaluation on five test subjects following four different predefined trajectories show that, in a typical indoor environment, WiPIHT achieves a median tracking error of less than 0.3 m in trajectory reconstruction.

The remainder of this paper is organized as follows:

In Section 2, we review related work. Section 3 provides an overview of the WiPIHT system. Section 4 introduces the data collection and noise separation preprocessing. Section 5 describes the feature extraction, including location-independent speed and direction features. Section 6 presents the methods for motion trajectory reconstruction, including initial position estimation and motion detection. Implementation, evaluation, and use cases are provided in Section 7. We conclude with a summary and discussion in Section 8.

## 2. Related Works

### 2.1. Non-RF-Based Human Activity Tracking

Non-RF-based human activity recognition and tracking primarily rely on optical [28,29,30,31] and acoustic signals. In recent years, most optical tracking research has been based on cameras or infrared, combined with algorithms for human identification. However, this approach has several drawbacks, such as the need for specialized equipment and hardware, significant environmental influence, and privacy concerns. Acoustically, most tracking uses ultrasound characteristics for recognition and tracking, but this method also suffers from hardware issues and unsatisfactory recognition range. In contrast, our proposed system does not rely on specialized hardware, protects user privacy, withstands changes in environmental layout and lighting, and can be applied in large spaces.

### 2.2. RF-Based Human Activity Tracking

RF-based [32,33,34,35,36] wireless sensing has seen substantial research and development in recent years, with WiFi sensing being applied across various domains. Among these studies, significant progress has been made in using CSI for human activity and localization detection [37,38,39,40], such as posture recognition, trajectory tracking, gesture recognition, and identity recognition. In gesture recognition, Tan et al. proposed a fine-grained gesture recognition system using conventional WiFi networks [41]. By analyzing the unique patterns of CSI, they could sense and recognize fine hand movements without any sensors. For identity recognition, Zhang et al. introduced WiFi-ID [42], which analyzes the walking patterns contained in channel variations to identify individuals. In terms of localization, Zhou et al. correlated CSI fingerprints with the target’s location, building a CSI fingerprint database [43]. In this system, when new CSI fingerprints appear, they are matched against the database to determine the location. Although accurate, constructing the fingerprint database is a massive task, and each database is specific to a fixed environment, limiting its general applicability. Wang et al. proposed WiTrace [44], which, while able to achieve passive tracking, still relies on specialized equipment and external clocks. WiDraw [45], although it uses only commercial WiFi without additional devices, requires a large number of TXs and RXs, limiting its practical use. WiDar [19] uses fewer WiFi transceivers but relies heavily on a large number of pre-collected measurement data as a training set. If the positions of the transceivers or other environmental layouts change, the original training set becomes invalid. Critically, many similar methods require precise information about transceiver positions and specific initial conditions, which is impractical for real-world applications. In contrast, our method uses only three commercial WiFi devices, without the need for data collection or additional training, achieving high-precision real-time passive tracking of human activities.

Table 1 shows a systematic comparison of WiPIHT and state-of-the-art WiFi trajectory reconstruction techniques across five critical dimensions: TX-RX positioning constraints, training data volume, hardware specifications, real-time performance, and initial position dependency. The comparative analysis reveals that WiPIHT demonstrates significant advantages including deployment flexibility (eliminating TX-RX positional constraints), training-free implementation, low cost efficiency, real-time processing, and initial position independence. These collective strengths collectively establish WiPIHT’s superior practical deployment value over existing methodologies.

## 3. System Overview

**Figure 1:**
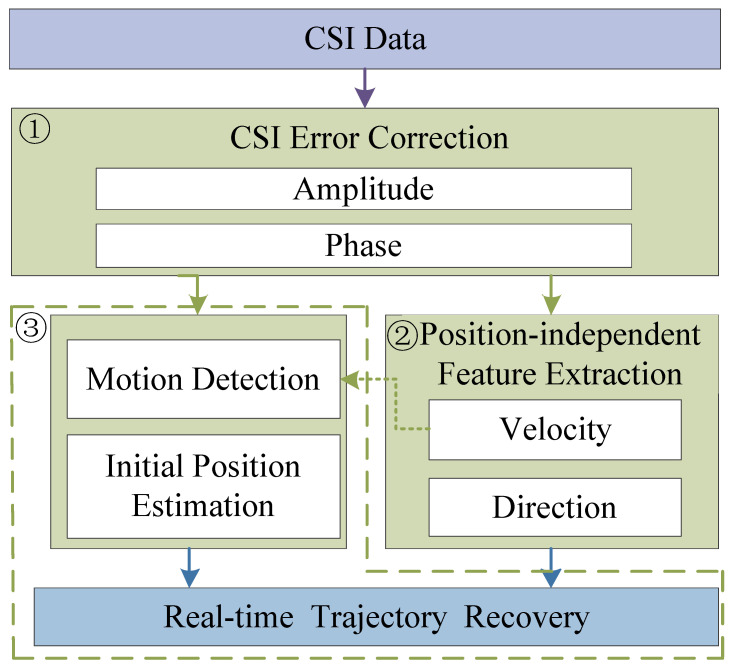
System overview.

In this study, we establish a real-world scenario where individuals are located indoors within the coverage of WiFi signals, and the Channel State Information of the WiFi is influenced by human activities. Our main objective is to utilize the CSI data received in real time by the WiFi RX to extract features that represent the real-time walking direction and speed of the humans. Additionally, we aim to calculate the initial position for each movement segment and determine the initial time of actions using motion detection. Subsequently, we track the indoor human positions and reconstruct the complete movement trajectory based on these features and positional information. Figure 1 provides a schematic diagram of our proposed system, which includes three core components: CSI error correction, extraction of position-independent speed and movement direction features, and real-time tracking and recovery of indoor movement trajectories.

CSI Error Correction. The purpose of this component is to perform the necessary preprocessing of the collected CSI data to correct errors that occur during data collection. During the data collection process, we use a WiFi router as the signal TX and two laptops equipped with WiFi signal reception capabilities as RXs. For error correction, we utilize the CSI ratio principle, employing methods such as amplitude ratio and phase difference to eliminate errors. Additionally, we apply S-G filtering and PCA to separate the effective components of the CSI stream that contain location, speed, and direction features.

Position-Independent Feature Extraction. The main objective of this component is to analyze the noise-separated CSI time series to extract features representing location-independent indoor human movement speed and direction. For speed features, we analyze the CSI autocorrelation function to obtain instantaneous movement speed characteristics of humans at any position within the monitored area. For direction features, we employ a movement angle detection method based on the Fresnel zone principle. By using the speed components generated by the Fresnel zones created by two intersecting links, we extract location-independent movement direction features without needing prior knowledge of the specific positions of the WiFi TXs and RXs.

Real-Time Trajectory Recovery. This part of our system is used to reproduce the target movement trajectory based on the dual features of speed and direction. It includes three components: initial position estimation, motion detection, and trajectory reconstruction. We estimate the initial position of the person by combining the multipath classification algorithm with the initial position of the TX-RX link and using triangulation. We estimate the start and end times of each movement segment by applying a sliding window to the speed–time waveform. Finally, by integrating the speed and direction feature curves and combining them with the initial position, we reconstruct and generate the person’s movement trajectory indoors.

## 4. CSI Error Correction

**Figure 2:**
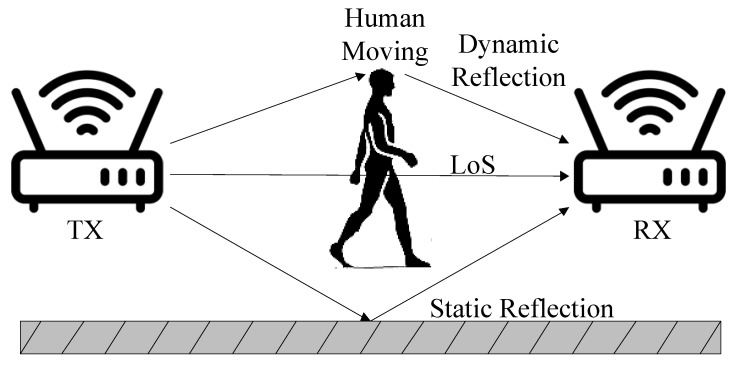
CSI multipath.

Commercial Off-The-Shelf (COTS) WiFi devices nowadays come equipped with multiple transmitting and receiving antennas [46]. These antennas form channels between each pair of transmitting–receiving antennas (TX-RX antenna pairs) through multiple subcarriers. The state of these channels is intuitively quantified by Channel State Information values, which essentially represent the channel frequency response of each subcarrier between each TX-RX antenna pair. The signal received by the receiving antenna is essentially the result of multiple multipath signals interfering with each other in the surrounding environment. The movement of a human being affects the distribution of the electromagnetic field in space, leading to changes in signal propagation paths and phase delays. Therefore, we can extract motion changes by analyzing the Channel State Information. These changes are effectively reflected in the CSI values of the subcarriers transmitted between each TX-RX antenna pair and are used to identify indoor activity trajectories. As shown in Figure 2, all TX-RX propagation paths can be divided into static paths and dynamic paths. Static paths are composed of the Line of Sight (LoS) and other static object reflections, which can be considered constant because they remain unchanged. Dynamic paths are reflected by moving objects, so the CSI can be expressed as follows (1):

(1) $$\begin{array}{c} H\left(f,t\right)=H_{s}\left(f,t\right)+H_{d}\left(f,t\right)=H_{s}\left(f,t\right)+A\left(f,t\right)e^{-j2\pi \frac{d(t)}{\lambda }} \end{array}$$

Here, $H_{s}\left(f,t\right)$ represents the composite CSI of static paths, $H_{d}\left(f,t\right)$ represents the composite of dynamic paths, λ is the wavelength, and A(f,t), *e*, and d(t) represent the attenuation, phase shift, and path length of $H_{d}\left(f,t\right)$, respectively.

### 4.1. Fresnel Zone and CSI Phase Rotation

**Figure 3:**
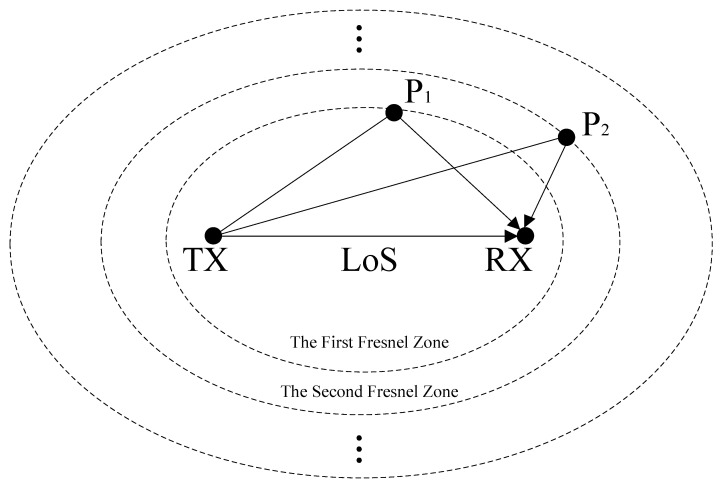
Fresnel zone.

The Fresnel zone [47,48] is a concept that was proposed to study the interference and diffraction of waves, revealing the physical characteristics of wave propagation from the source to the observation point. It is manifested as a series of concentric ellipses with the TX-RX pair as the foci based on the different path lengths of the wave propagation path. As shown in Figure 3, for a given wireless signal with wavelength λ, assuming TX-RX is a pair of wireless RF transceiver devices, the Fresnel zone can be described by the following Formula (2):  

(2) $$|TXP_{n}\left|+\right|P_{n}RX|-LoS=n\lambda /2$$

**Figure 4:**
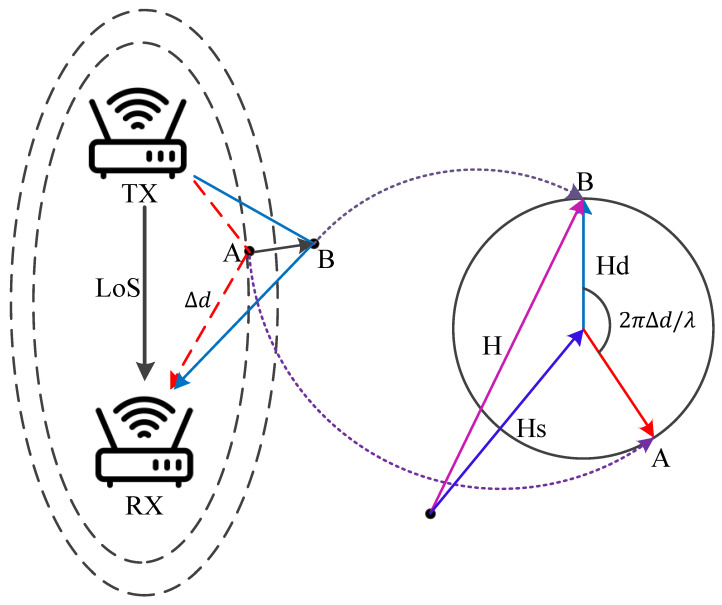
Fresnel zone and CSI dynamics.

The Fresnel zone theory waveform reveals a phenomenon: the impact of different motion modes at different positions on the CSI depends on how many Fresnel zone layers the target has crossed during movement, as well as the target’s relative position and direction with respect to the TX-RX within the Fresnel zone. As the target moves, the reflection path length changes, and the dynamic component $H_{d}\left(f,t\right)$ rotates accordingly. If the change in reflection path length is less than one wavelength, the superimposed CSI varies along the arc. For each Fresnel zone layer the target passes through, the reflection path changes by λ, and the dynamic phase changes by 2π. Therefore, when the dynamic path propagates along a reflection path of length *d*, the phase rotation is 2πd/λ, as shown in Figure 4. We use the dynamic phase change of CSI to reconstruct the target’s movement trajectory.

### 4.2. CSI Error Correction and Denoising Based on Amplitude and Phase

**Figure 5:**
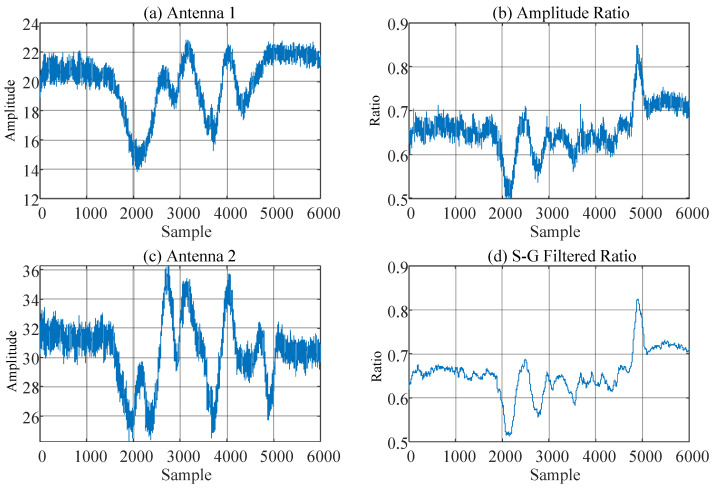
Amplitude noise reduction.

In practical applications, CSI contains a large number of errors in both amplitude and phase, making it unsuitable for direct use. The main source of errors in amplitude is the Automatic Gain Controller (AGC) in WiFi NICs [49,50]. Due to the uncertainty in the AGC power control and surrounding electromagnetic noise, the amplitude fluctuates. These noise-induced fluctuations are almost consistent across different receiving antennas of the same RX. Therefore, we use the amplitude ratio between adjacent antennas to eliminate errors and retain the required components, as shown in Figure 5. Figure 5a,c show the amplitude variations of a subcarrier on two different antennas of the same RX when a person walks near the WiFi transceiver, including high pulses and burst noise. Figure 5b,d show the amplitude ratio of the two antennas after noise elimination and filtering for this subcarrier.

For the dynamic phase of the CSI, the sampling frequency and center frequency of the TX-RX are not exactly the same, leading to Channel Frequency Offset (CFO) and Sampling Frequency Offset (SFO) [51,52], which severely distort the phase information of the CSI. To address this issue, we use the CSI commercial model [53,54,55,56] to correct the errors. Assuming there is a TX-RX pair at two fixed positions and a moving target, the signal from the LoS and static objects in the reflection environment forms the static component, while the target’s reflection forms the dynamic signal component. The form of the CSI commercial model is as follows (3):

(3) $$\begin{array}{cc} H_{q}\left(f,t\right) & =\frac{A_{noise}\left(f,t\right)e^{-j\theta_{offset}\left(f,t\right)}\left(H_{s1}\left(f,t\right)+H_{d1}\left(f,t\right)\right)}{A_{noise}\left(f,t\right)e^{-j\theta_{offset}\left(f,t\right)}\left(H_{s2}\left(f,t\right)+H_{d2}\left(f,t\right)\right)}\\ \; & =\frac{H_{s1}\left(f,t\right)+H_{d1}\left(f,t\right)}{H_{s2}\left(f,t\right)+H_{d2}\left(f,t\right)} \end{array}$$

where $A_{noise}$ is the pulse noise in the amplitude of the CSI, and $\theta_{offset}$ is the random phase offset, like the CFO. $H_{s1,2}\left(f,t\right)$ and $H_{d1,2}\left(f,t\right)$ are the static and dynamic components of the RX’s two antennas, respectively. When the target moves a short distance, it can be assumed that the amplitude of the static component and the channel dynamic component remains unchanged on the two antennas of the RX, and the change in the arrival angle of the reflected signal from the moving target is very small. Therefore, the above equation can be simplified to the following (4):

(4) $$\begin{array}{c} H_{q}\left(f,t\right)=\frac{H_{s1}\left(f,t\right)+A_{1}\left(f,t\right)e^{-j2\pi \frac{d_{1}\left(t\right)}{\lambda }}}{H_{s1}\left(f,t\right)+A_{2}\left(f,t\right)e^{-j2\pi \frac{d_{2}\left(t\right)}{\lambda }}}=\frac{az+b}{cz+d} \end{array}$$

In this case, we take *z* = $e^{-j2\pi \frac{d_{2}\left(t\right)}{\lambda }}$, where *a*, *b*, *c*, and *d* are all constant complex numbers.

**Figure 6:**
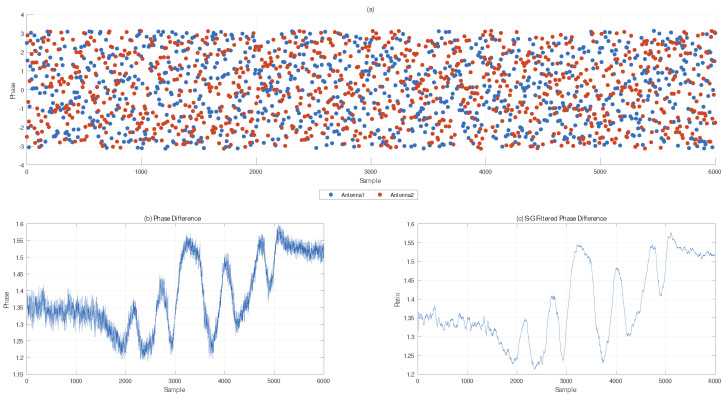
Phase noise reduction.

To address the scattering of original samples and the difficulty in extracting rotation angles on the complex plane of the CSI commercial model, we use an S-G filter to smooth the samples of the CSI commercial model. The result is shown in Figure 6. By performing PCA on all subcarriers and retaining the first PCA component, we further process the noise. Figure 6a shows the phase distribution of a subcarrier for two antennas on the same RX. Figure 6b,c depict the shape of the phase difference after noise elimination and filtering.

Compared to traditional noise reduction methods such as low-pass filtering [57], using the amplitude ratio with the CSI phase has significant advantages in error correction for CSI time series. Low-pass filtering methods cannot eliminate CFO errors that change over time and can only remove SFO. Using the CSI phase and amplitude ratio can first remove errors from the AGC, CFO, and SFO in the signal, suppress pulse noise, eliminate random offsets, restore relatively accurate CSI in the complex plane, and remove error components that cannot be removed by conventional methods. Secondly, PCA can remove subcarrier components of CSI that are not highly correlated with human walking, reducing the computational complexity by reducing the dimensionality of the CSI contained in subcarriers, and avoiding using a large amount of irrelevant subcarrier information for correlation calculations. These two advantages are also one of the reasons for the high accuracy of our WiPIHT indoor mobile trajectory reconstruction. Beyond high ambient noise, signal strength constitutes another critical factor influencing the operational effectiveness of the system. Our findings indicate that, under typical indoor WiFi conditions (ranging from −40 dBm to −60 dBm), the system sustains stable operation. However, under severely compromised signal conditions (e.g., <−70 dBm), tracking accuracy is demonstrably compromised. Therefore, to guarantee consistent system efficacy, proactive signal strength monitoring is recommended during deployment, ensuring operation within this nominal WiFi signal intensity range.

## 5. Position-Independent Feature Extraction

### 5.1. Position-Independent Real-Time Velocity

Estimating location-independent passive real-time walking speed from wireless signals is highly challenging. To address this, we treat environmental objects as multiple scatterers and propose a real-time speed estimation method based on the autocorrelation function. By considering multiple scattering paths and utilizing the statistical properties of these scattering paths rather than relying on position, speed direction, or environmental conditions, we derive the moving speed from the ACF. Due to the multipath effect, a human in the WiFi signal coverage area is regarded as a collection of multiple scatterers [23], reflecting signals in different directions and combining with signals scattered by other objects through multipath propagation. The CSI signal can be decomposed into a combination of static scattering, dynamic scattering, and noise, as shown in Equation (5):

(5) $$H\left(t,f\right)=\sum_{i\in Vs(t)}H_{i}\left(t,f\right)+\sum_{j\in Vd(t)}H_{j}\left(t,f\right)+\varepsilon \left(t,f\right)$$

where H(t,f) represents the total CSI signal at time *t* and frequency *f*, Vs(t) represents the collection of static scatterers, Vd(t) represents the collection of dynamic scatterers, $H_{i}\left(t,f\right)$ represents the contribution of the *i*-th static scatterer at time *t* and frequency *f*, and ε(t,f) is the noise term. In a very short period of time, it can be reasonably assumed that Vs and Vd are approximately constant. Dynamic reflections are dominated by the movement of the human body, and, therefore, the autocorrelation function of H(t,f) can be expressed as follows (6):

(6) $$r^{H}\left(t,f\right)=a\left(f\right)J_{0}\left(kvt\right)+n\left(t,f\right)$$

where a(f) is the signal gain at subcarrier frequency *f*, which can be obtained through measurement, $J_{0}$ is the zero-order Bessel function, *k* is a proportionality constant, *v* is the instantaneous walking speed, and n(t,f) is the noise term, usually Gaussian white noise.

**Figure 7:**
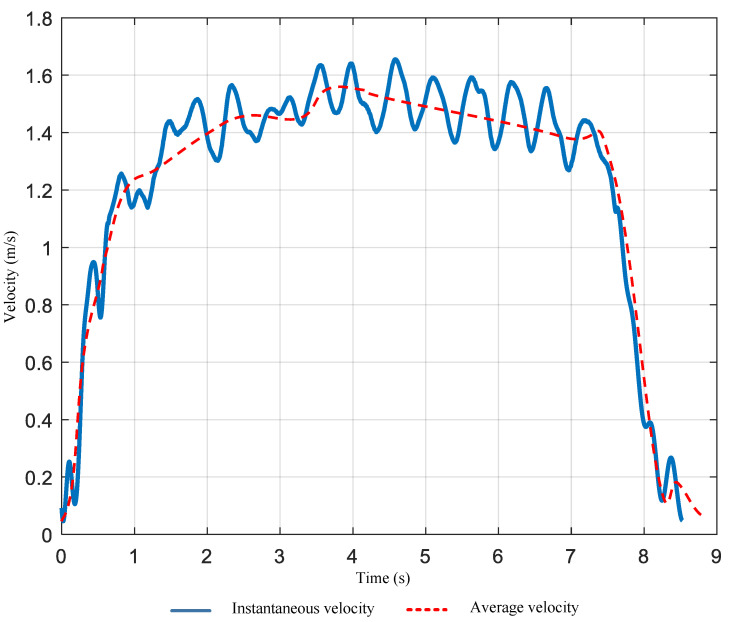
Velocity–time curve.

Next, the maximum ratio of velocity signals on each subcarrier is combined as follows (7):

(7) $$S\left(t\right)=\sum_{f\in F}w\left(f\right)r^{H}\left(t,f\right)$$

where S(t) is the combined velocity signal, and w(f) is the combined weight of the subcarriers at frequency *f*. When sampling rate $F_{s}$ is high enough, $\hat{w}\left(f\right)={\hat{r}}^{H}\left(t=\frac{1}{F_{s}},f\right)$. The waveform of the composite velocity signal exhibits correspondence with the Bessel function $J_{0}\left(kvt\right)$. Consequently, the velocity *v* can be derived by matching their key characteristic points as follows (8):

(8) $$\hat{v}=\frac{x_{0}}{k\hat{t}}=\frac{x_{0}\lambda }{2\pi \hat{t}}$$

where $x_{0}$ is the mathematical constant corresponding to the first peak of the Bessel function, and $\hat{t}$ denotes the time delay precisely aligned with the first peak in the composite velocity signal. Using this, we can calculate the instantaneous speed *v* at each time point during human movement. The human movement velocity curve in an indoor space is shown in Figure 7. Since the instantaneous walking speed of a human cyclically increases and decreases, we take its average real-time velocity curve. The blue solid line represents the instantaneous speed, while the red dashed line represents the average speed, which follows the overall trend of the instantaneous speed.

### 5.2. Position-Independent Real-Time Motion Direction

For sensing the direction of the target movement, most current methods cannot avoid relying on specific information such as the device position or the initial position of the person. In some environments, these methods are significantly limited. We adopt a position-independent method for determining human movement direction. The core idea is not to determine the absolute direction of movement based on the relative position of the transceivers, but rather to judge the current movement direction relative to the previous movement direction [58]. In this way, when the speed–time curve is constant, the overall trajectory shape of human movement remains relatively fixed regardless of how it is rotated. No matter how the transceivers are arranged, for the same real movement path, the overall trend of trajectory angle changes remains consistent. For trajectory estimation in a two-dimensional plane, at least two pairs of interacting transceivers are needed to estimate changes in the target movement direction.

**Figure 8:**
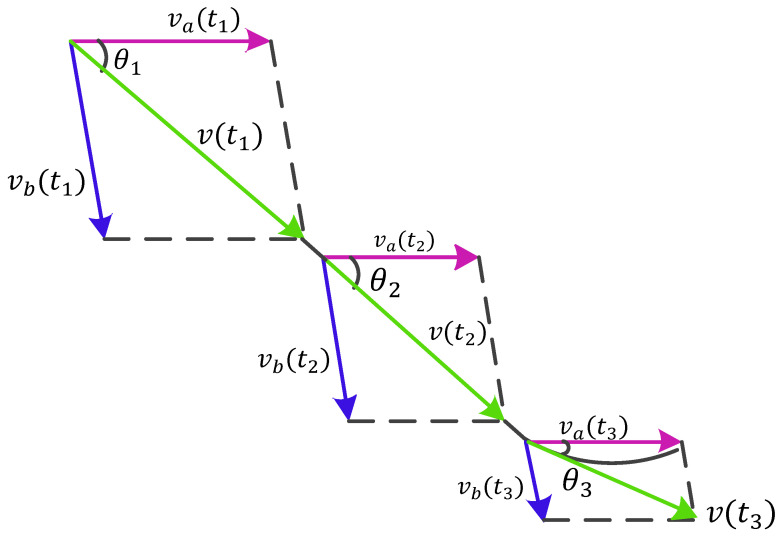
The velocity component in movement direction.

We know that any vector in a plane can be decomposed into two different directional vectors. Therefore, a real-time motion vector with velocity can also be decomposed into two velocity components in different directions. In the directional decomposition within the Fresnel zone model, the instantaneous motion direction is primarily governed by the normal-velocity components along two intersecting Fresnel zone boundaries. These velocity-induced variations are distinctly manifested through dynamic phase rotations in the CSI. Consequently, directional changes can be quantitatively determined by calculating the relative vector shift between the current motion direction and the preceding trajectory segment. As shown in Figure 8, when the target’s movement direction changes, that is, when the direction of velocity *v* changes, the ratio of the two fixed directional velocity components also changes. Conversely, when the direction of velocity *v* is unchanged, the ratio of the two velocity components remains constant. For example, at different times $t_{1}$ and $t_{2}$, the magnitudes of instantaneous velocities $v(t_{1})$ and $v(t_{2})$ may differ, but, if their directions are consistent, the ratio of their components c and $v_{a}$ does not change. Through geometric analysis, θ can be expressed by Equation (9):

(9) $$\theta =arctan\frac{\frac{|V_{b}|}{|V_{a}|}-cos\alpha }{sin\alpha }$$

Here, α is the constant angle between the two velocity components, so θ only depends on the ratio $v_{b}/v_{a}$. Consequently, θ is determined solely by the changes in the direction of *v*. Although *v* can be decomposed into any two non-parallel components, the directions of these components should remain fixed during the target’s movement.

**Figure 9:**
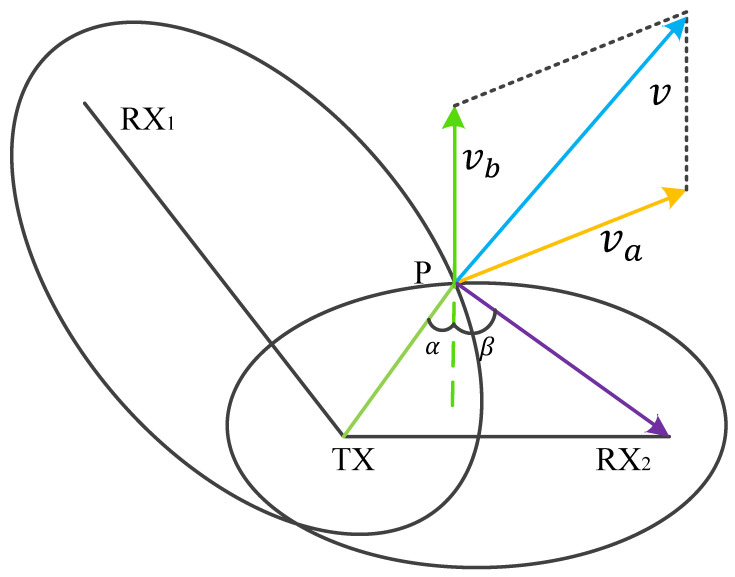
The velocity component in the Fresnel zone.

As shown in Figure 9, for any two non-parallel TX-RX links, we obtain their intersecting Fresnel zones. It has been demonstrated by scholars that the velocity component in the direction of the Fresnel zone normal is the main factor affecting WiFi signal changes. Therefore, we decompose the target’s real-time directional velocity components $v_{a}$ and $v_{b}$ into the normal directions of the two Fresnel zone boundaries. Within a very short time span, the distance moved by the target human is much less than the LoS, so the angle between $v_{a}$ and $v_{b}$ can be considered constant. Thus, the only factor reflecting the target human’s movement direction is the ratio of the two components.

When we do not know the exact orientation of the human body relative to the TX-RX, we cannot calculate the specific values of $v_{a}$ and $v_{b}$. To solve this problem, we need to find other parameters that can reflect the ratio of the velocity components. From Figure 9, we can see that, within a very short time interval Δ*t*, only $v_{b}$ in the Fresnel zone, formed by the TX-RX_2_ link, causes a change in the reflected path of Δ*d*. Since *v_b_* is in the direction of the normal to the ellipse, and Δ*d* is much smaller than the LoS, *α* and *β* can also be considered constant. Therefore, the distance change of TX-P is $v_{b}cos\alpha \Delta t$, and the distance change of P-RX_2_ is $v_{b}cos\beta \Delta t$. The change in velocity $\Delta v_{b}$ is as follows (10):

(10) $$|\frac{\Delta d}{\Delta t}\left|=\right|V_{b}|\left(cos\alpha +cos\beta \right)$$

Similarly, for another non-parallel Fresnel zone, we can determine the change in the $\Delta v_{a}$ velocity component over time Δt. Since α and β are considered fixed values, we know from the previous equation that $\Delta v_{b}/\Delta v_{a}$ is equal to $\Delta d_{b}/\Delta d_{a}$, where $\Delta d_{b}$ and $\Delta d_{a}$ are the changes in the reflected path lengths within their respective Fresnel zones for TX-RX_2_ and TX-RX_1_ over Δt. In the time series, this ratio curve can reflect the change in the overall direction angle. If we denote the direction angle change curve at time *t* relative to *t*-Δt as θ(t), then we have the following (11):

(11) $$\theta \left(t\right)=arctan\frac{\Delta d_{2}\left(t\right)}{\Delta d_{1}\left(t\right)}$$

Based on the relationship between the CSI phase rotation and Fresnel zones discussed earlier, we can determine the reflected path Δd(t) as follows (12):

(12) $$\Delta d\left(t\right)=\frac{\lambda }{2\pi }∠\Delta H\left(f,t\right)$$

Thus, θ(t) can be expressed as follows (13):

(13) $$\theta \left(t\right)=arctan\frac{∠\Delta H_{2}\left(f,t\right)}{∠\Delta H_{1}\left(f,t\right)}$$

where $∠H_{2}\left(f,t\right)$ and $∠H_{1}\left(f,t\right)$ are the dynamic phase rotation angles of the CSI on two RXs. In this way, we can obtain the curve of the directional angle change on the time series in any two non-parallel TX-RX links without the need to know the specific position of the target human body relative to the WiFi device. Next, we represent the time series *T* as a set of Δt, with specific Δt selected based on experience and different situations. The directional change curve M(t) can be represented as follows (14):

(14) $$M\left(t\right)=\sum_{i=1}^{t}\theta \left(i\right),t\in \left[1,T/\Delta t\right]$$

**Figure 10:**
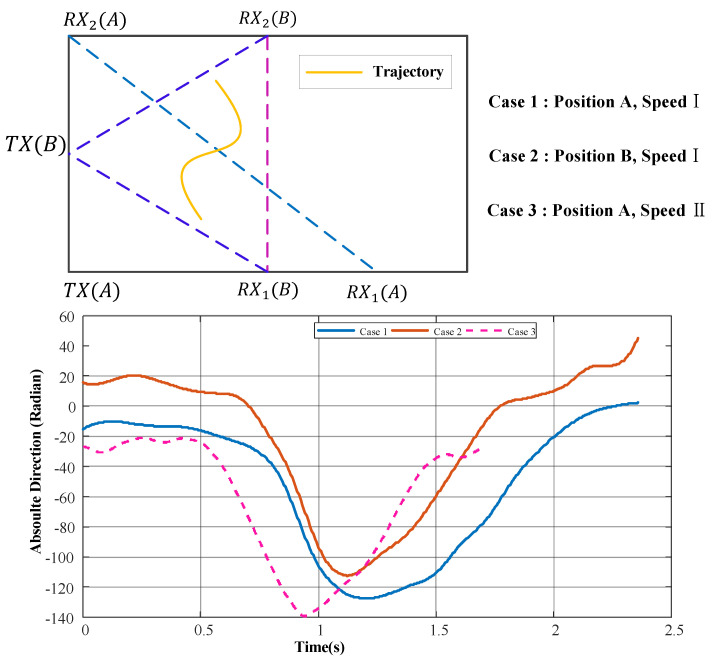
Direction change in three cases.

Figure 10 shows the direction change curves for the same moving trajectory of the target human body relative to different TX-RX positions and moving speeds. It can be seen that, regardless of the positions of the TXs and RXs, or the initial point and speed of the target human body in space, the direction curves all exhibit the same trend of change.

## 6. Real-Time Trajectory Recovery

### 6.1. Motion Detection

**Figure 11:**
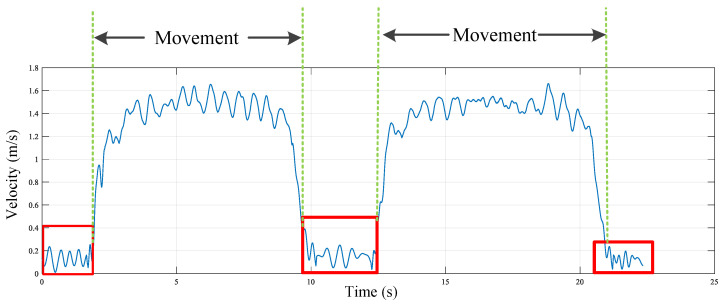
Motion detection.

Since human movement in indoor spaces may not be continuous, to avoid errors when the target is stationary, we need to introduce a motion detection mechanism. To do this, we use a sliding window to detect the known real-time speed curve. Only when continuously stable fluctuations matching the normal human movement speed are detected do we judge that the target is in a moving state. The sliding window is set to 0.2 s with a 50% overlap to detect real-time average speed changes. We use the empirical threshold of human walking speed to detect the start and end time points of each movement. To match the speed curve reasonably with the direction change curve, we also segment the speed curve in time series *T* using Δ*t*. As shown in Figure 11, we match the detected start and end time points of the sliding window with the time series set *T*. By deleting the non-motion time nodes between two movements, we obtain a motion speed curve without pauses.

### 6.2. Initial Position Estimation

Through the human velocity and direction change curves, we can obtain the relative trajectory shape. However, the absolute trajectory is still influenced by the initial position and initial angle. Therefore, in practical applications, it is still necessary to correct the relative trajectory based on the position of the human body relative to the TXs and RXs. To obtain the initial position, we use the MUSIC algorithm [20,25,26] to calculate the arrival angle of the incident signal. The basic idea is that incident signals from different angles will introduce different phase changes between the receiving antennas. We use the reflection of the human body as the signal source, the RX receives the reflected signal, and we estimate the initial position in conjunction with the triangulation. We analyze the eigenstructure of the correlation matrix $R_{X}$ of the signal *X* from the received CSI data as follows (15):

(15) $$\begin{array}{cc} R_{x} & =E[XX^{H}]\\ \; & =AE\left[SS^{H}\right]A^{H}+E\left[NN^{H}\right]\\ \; & =AR_{s}A^{H}+\sigma^{2}I \end{array}$$

where *A* is the overall steering matrix, *S* is the path signal at the antenna, *N* is Gaussian noise with a zero mean and variance $\sigma^{2}$, $X^{H}$ is the conjugate transpose of *X*, $R_{S}$ is the source correlation matrix, and *I* is the identity matrix. *E* is the matrix of eigenvectors corresponding to the eigenvalues of the correlation matrix $R_{X}$, and, based on the size of the eigenvalues, the eigenvectors are divided into the signal subspace and the noise subspace. The smallest *K* eigenvectors corresponding to the smallest eigenvalues form the noise subspace $E_{N}=\left[e_{1},e2,\ldots ,e_{K}\right]$, and the remaining eigenvectors form the signal subspace $E_{S}=\left[e_{k+1},e_{k+2},\ldots ,e_{M}\right]$, where *M* is the total number of eigenvectors. The signal subspace and the noise subspace are orthogonal. For each possible target position, we calculate the MUSIC spectrum through the direction angle θ and the distance *d* as follows (16):

(16) $$P_{MUSIC}\left(\theta ,d\right)=\frac{1}{a^{H}\left(\theta ,d\right)E_{N}E_{N}^{H}a\left(\theta ,d\right)}$$

where a(θ,d) is the array manifold vector, representing the array response to a specific direction angle and distance. By locating the peak in the MUSIC spectrum, we can calculate the initial position of the target corresponding to the direction angle and distance.

**Figure 12:**
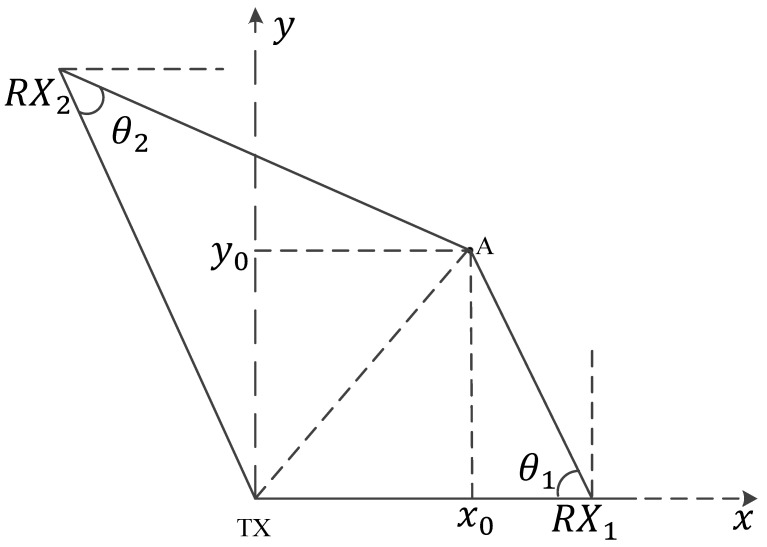
Initial position estimation.

As shown in Figure 12, when the TX is the origin and the coordinates of the RXs are ($X_{RX1}$, $Y_{RX1}$) and ($X_{RX2}$, $Y_{RX2}$), respectively, we solve for the dynamic path and the AoAs $\theta_{1}$ and $\theta_{2}$ for the LoS on the RXs. The initial coordinates ($x_{0}$, $y_{0}$) are determined through triangulation as follows (17):

(17) $$\begin{array}{c} x_{0}=\frac{y_{RX_{2}}+tan\left(\theta_{1}\right)x_{RX_{1}}-tan\left(\theta_{2}\right)x_{RX_{2}}}{tan\left(\theta_{1}\right)-tan\left(\theta_{2}\right)}\\ y_{0}=tan\left(\theta_{1}\right)\left(\frac{y_{RX_{2}}+tan\left(\theta_{1}\right)x_{RX_{1}}-tan\left(\theta_{2}\right)x_{RX_{2}}}{tan\left(\theta_{1}\right)-tan\left(\theta_{2}\right)}-x_{RX_{1}}\right) \end{array}$$

There are two initial positions: the first is the initial position of the TX and RX, and the second is the initial position of the human body in the room. For the TX and RX, since WiPIHT relies on the dynamic phase changes generated by the movement of the human body in the cross Fresnel zone to determine the direction change, this results in the two TX-RX links not being parallel, and a certain angle needs to exist. The closer the angle is to 90 degrees, the more accurate the judgement will be. This conclusion will be demonstrated in subsequent experiments. Regarding the estimation of the initial position of the human subject, when there are only two TX-RX links, if the human body is initially in any one of the links and moves along the link, it is difficult to determine the initial position, which is an inherent limitation of the MUSIC algorithm. Despite this limitation, our system inherently preserves the capability to reconstruct the shape of movement trajectories even without prior knowledge of the initial position. When the initial position is required, the ambiguity in the AoA estimation can be mitigated by deploying additional cooperative RX units. Crucially, when a subject moves along one TX-RX link, the remaining active links maintain continuous tracking capability, simultaneously enhancing system accuracy and robustness through redundant measurements.

Once the relative trajectory and initial coordinates are determined, the initial rotation angle $\theta_{0}$ that needs to be corrected can be determined through the positions of the TXs and RXs. This corrects the direction change curve to M(t) = M(t) + $\theta_{0}$.

### 6.3. Trajectory Recovery

To reconstruct the path coordinates of human movement, we use the following approach: Assuming the target’s speed at time *t* is V(t), the moving direction angle is M(t), and the initial point coordinate is ($x_{0}$, $y_{0}$), the calculation process first decomposes the velocity V(t) into components in the x and y directions based on M(t) (18):

(18) $$\begin{array}{c} V_{x}\left(t\right)=V\left(t\right)cos\left(\frac{M(t)\pi }{180}\right)\\ V_{y}\left(t\right)=V\left(t\right)sin\left(\frac{M(t)\pi }{180}\right) \end{array}$$

For any time *t*, the path coordinates (x(t), y(t)) can be obtained by integrating the velocity components. The formula for the target’s path at time t is obtained as follows (19):

(19) $$\begin{array}{c} \begin{array}{c} x\left(t\right)=x_{0}+\int_{0}^{t}V_{x}\left(t\right)dt=x_{0}+\int_{0}^{t}V\left(t\right)cos\left(\frac{M(t)\pi }{180}\right)dt\\ y\left(t\right)=y_{0}+\int_{0}^{t}V_{y}\left(t\right)dt=y_{0}+\int_{0}^{t}V\left(t\right)sin\left(\frac{M(t)\pi }{180}\right))dt \end{array} \end{array}$$

**Figure 13:**
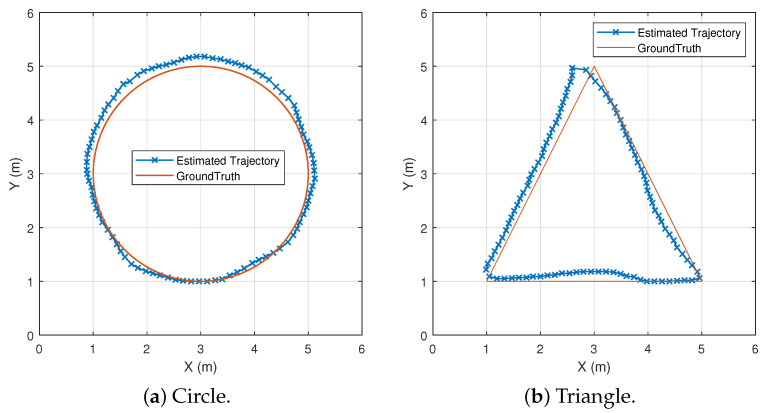
Trajectory recovery.

**Figure 14:**
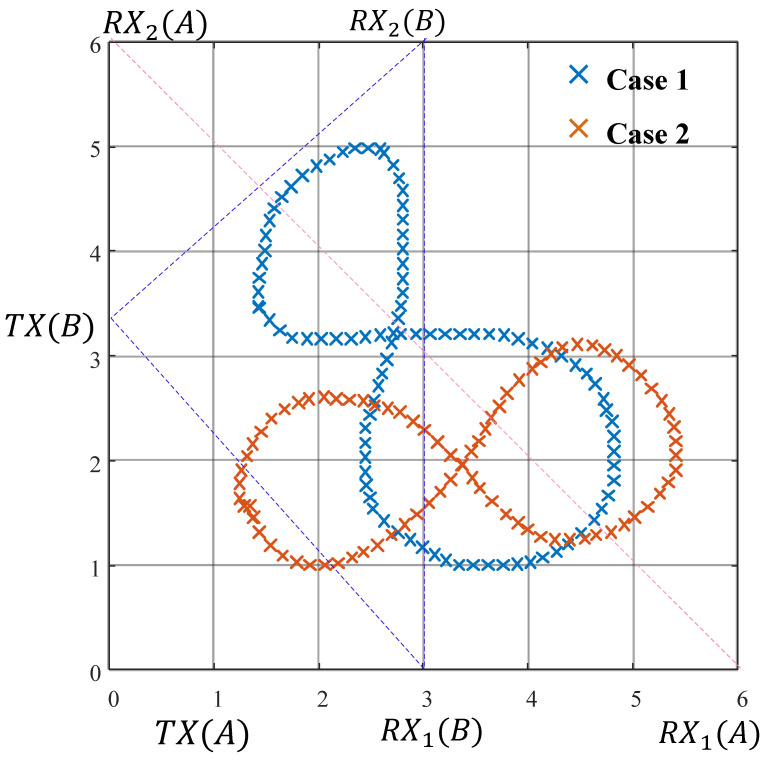
Trajectory recovery for different transceiver arrangements.

Thus, within the time series *T*, the target moves smoothly in the plane. Figure 13 shows the preset trajectory with Δ*t* of 0.1s and the corresponding reconstructed path point coordinates and a smooth trajectory. Figure 14 shows the reconstructed shapes of the moving trajectories for the same path with different TX-RX positions and unknown positions. It can be observed that, even if the initial position and the positions of the TXs and RXs are unknown, the shapes of the moving trajectories for the same path are approximately the same. The pseudocode for this part is shown in Algorithm 1.
**Algorithm 1:** WiPIHT Trajectory Recovery
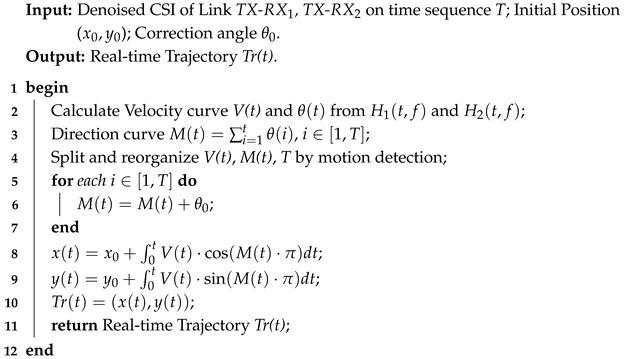


The trajectory fusion algorithm fundamentally operates by integrating the derived time–velocity and time–angle (directional) curves to compute time–position trajectories (paths). To mitigate the error accumulation inherent in prolonged integration, we implement a motion detection mechanism that segments continuous motion into discrete intervals. Each time motion cessation is detected, the system performs recalibration, preventing unbounded error propagation. Given our high sampling rate of 300 Hz and the typical trajectory duration of 1–2 min in our experiments, the integration-based approach consistently satisfies the required localization accuracy.

## 7. Experiment and Evaluation

### 7.1. Experimental Setup

**Figure 15:**
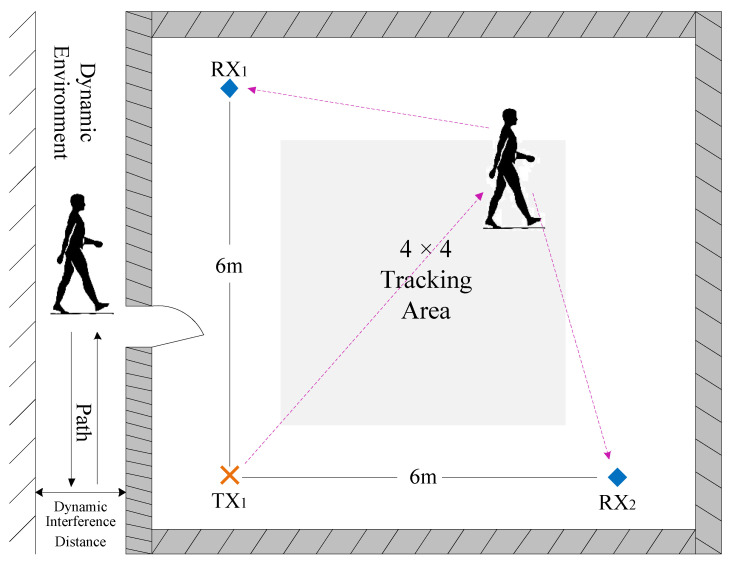
Experiment setup.

We used conventional commercial WiFi transceiver devices to implement WiPIHT. For the TX, we employed a TP-LINK TL-WDR5620 router equipped with a monopole antenna. The antenna featured a gain of 2 dBi and operated in the 2.4 GHz band. For the RX, we used a Dell Latitude E7470 laptop fitted with an Intel 5300 WiFi Network Interface Card. The laptop was configured with three external omnidirectional antennas, each with a gain of 3 dBi. The fixed antenna separation was set to single-wavelength spacing *λ*. All antennas were connected via SMA connectors. CSI was acquired using the Linux 802.11n CSI Tool, with CSI data from 30 subcarriers per TX-RX antenna pair collected at a sampling rate of 300 Hz. Signal processing employed a 64-point FFT (per IEEE 802.11n standard), followed by S-G filtering using a second-order polynomial and a 31-sample window (approximately 100 ms). PCA was then applied for denoising, retaining principal components accounting for >85% of the cumulative variance. We chose a classic architectural layout measuring 6.6 × 7.8 m as our experimental site. Figure 15 shows the overhead layout of the experimental area, with a Cartesian coordinate system constructed using the edges of the LoS of the two TX-RX pairs. This coordinate system was used as the reference for reconstructing the absolute movement trajectory in subsequent experiments. Considering the site conditions, we set up the TX and RX ends at the positions indicated in the figure, with TX coordinates at (0, 0), RX_1_ coordinates at (0, 6), and RX_2_ coordinates at (6, 0). The middle 4 × 4 range was the trajectory tracking and perception area. The TX used one transmission antenna (MT = 1), while the RX had three reception antennas (MR = 3), and each TX-RX antenna pair included three CSI data links. The TX and RXs were placed on desks 1 m above the ground, 6 m apart, with no other static objects in the environment.

We collected indoor movement data from five test subjects: three adult male subjects and two adult female subjects. During the data collection process, the test subjects walked naturally along the preset trajectories marked in the tracking area until the end of the trajectory. In the basic experiment, a total of four different sets of trajectories were tested, with each trajectory walked by each person five times. We evaluated the indoor movement trajectory reconstruction performance of the five test subjects using seven metrics: the trajectory reconstruction error, the impact of target movement speed, the impact of the static environment, the impact of the initial positions of the TXs and RXs, the impact of user diversity and preset trajectory diversity, the impact of the sampling rate, and the impact of other dynamic objects.

### 7.2. Experimental Results

#### 7.2.1. Trajectory Recovery Error

**Figure 16:**
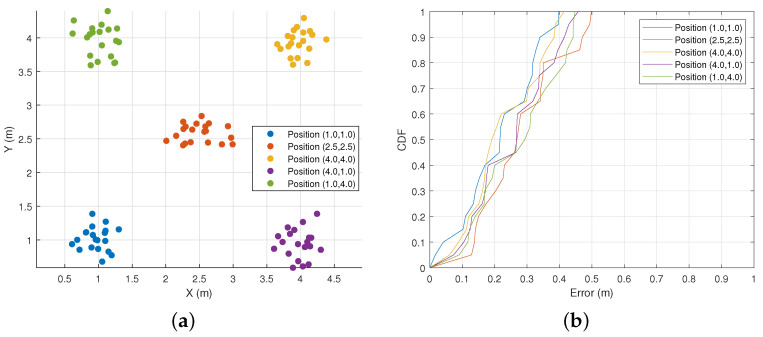
Initial position error. (**a**) Estimated position distribution. (**b**) Initial position error CDF.

Initial Position Error. First, we evaluated 100 sets of initial position data generated by all test subjects, with five preset initial positions: (1.0, 1.0), (1.0, 4.0), (4.0, 1.0), (4.0, 4.0), and (2.5, 2.5). The five test subjects started with the five preset initial positions and walked freely for a short distance, repeating each position four times. Figure 16a shows the distribution of the estimated initial position coordinates for each initial position. Figure 16b represents the degree of error between the estimated and actual positions, shown as a Cumulative Distribution Function (CDF). From the results, it can be seen that the estimated position error within the 90th percentile is within 0.47 m. This error is caused by the inherent error of the MUSIC algorithm itself and some weak dynamic interference. The experiment shows that the initial position estimation method used by WiPIHT can effectively complete the task.

**Figure 17:**
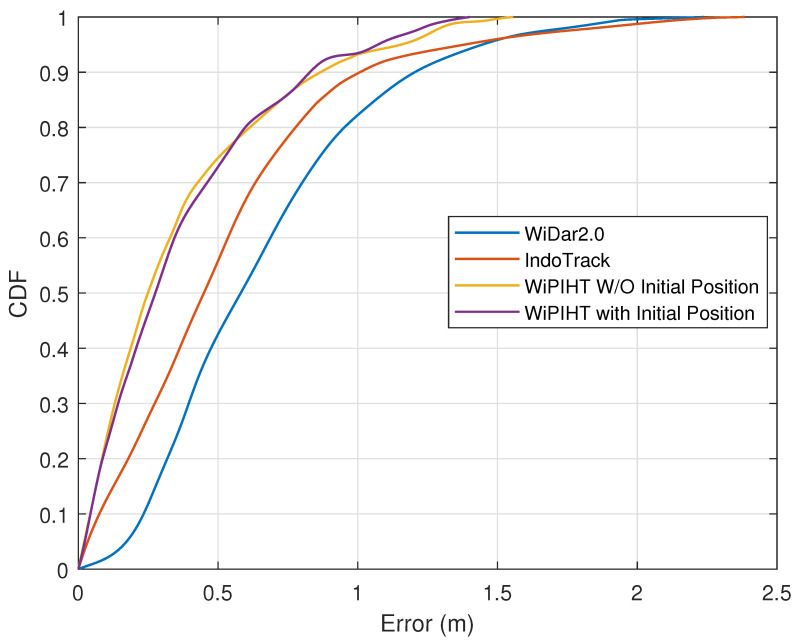
Two-dimensional trajectory error.

Two-Dimensional Trajectory Error. We evaluated the indoor trajectory reconstruction accuracy for 100 randomly generated preset trajectories in the experimental site, with each trajectory being uniformly distributed within the tracking area and including a combination of paths such as straight lines and curves. Figure 17 shows the CDF of the trajectory reconstruction errors for all preset trajectories on the 2D plane. It can be seen that the error within the 80th percentile can be controlled within 0.6 m, with the median error is kept below 0.28 m. The results also indicate that, when the initial positions of the TXs and RXs are unknown, the error in the reconstructed trajectory shape is almost the same as when these positions are known. When the initial position is unknown versus known, corresponding to the “WiPIHT W/O Initial Position” and “WiPIHT with Initial Position” cases, the comparable trajectory recovery errors between the two scenarios precisely demonstrate the position-independent nature of our method. This indicates that the path shape can be accurately recovered even without any prior knowledge of the initial position. We also compared WiPIHT with the more advanced IndoTrack [20] and WiDar2.0 [21] spatial positioning trajectory reconstruction methods. As shown in the figure, our approach significantly outperforms these two methods in terms of trajectory tracking error.

#### 7.2.2. Impact of Movement Speed

**Figure 18:**
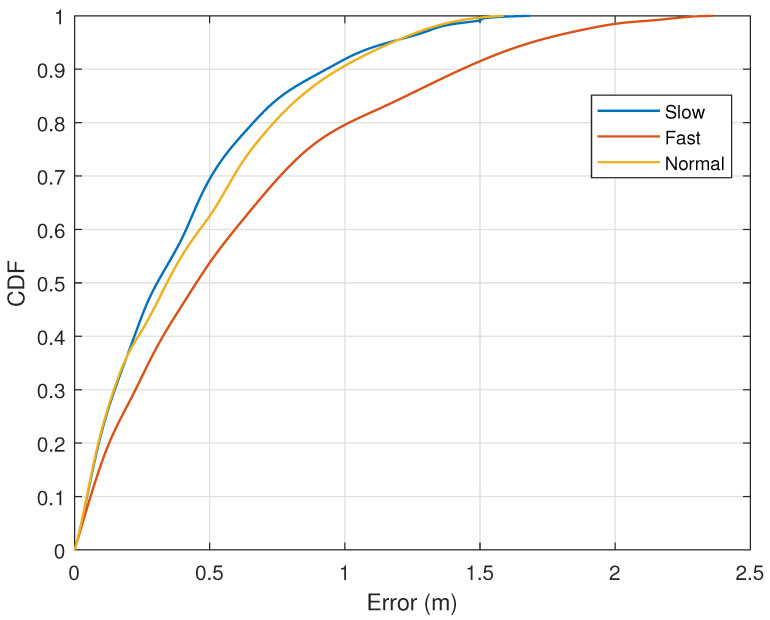
Error and CDF for three speeds.

To demonstrate the impact of different movement speeds of the test subjects on the experimental results, we asked the test subjects to perform five repeated experiments on the preset trajectory at three different speeds. We calculated the average speed of each movement by measuring the time taken to complete a trajectory. The three speeds were categorized as low speed (0.5–1.0 m/s), medium speed (1.0–1.6 m/s), and high speed (1.6–2.5 m/s). Figure 18 shows the trajectory reconstruction errors under the three different speed ranges. We can see that the median error for all three speeds is within 0.45 m. However, the error increases significantly when the speed exceeds 1.6 m/s. This increase is mainly due to the instability of the test subjects’ posture during fast movements, which made it difficult for them to be considered a single scatterer, leading to unstable instantaneous speed estimation. The figure demonstrates the tracking accuracy under different velocities. It can be observed that the curves for low and medium velocity are closely aligned, demonstrating that the system maintains stable performance within the typical operating speed range. This low discrepancy in the error presents exactly as expected and directly manifests the robustness of our approach.

#### 7.2.3. Impact of Static Layout

**Figure 19:**
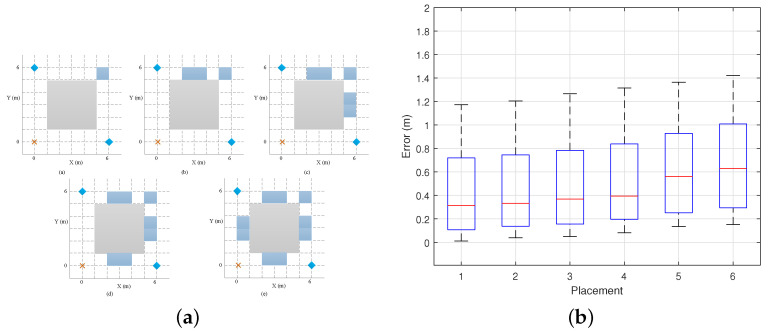
Comparison of different static environments. (**a**) Static environment layout. (**b**) Impact of static layout.

To investigate the impact of static environmental layouts, we conducted experiments in five additional different scenarios. Under a fixed preset trajectory, the experiments were carried out with the environmental layouts shown in Figure 19a. The crosses represent the TX positions, the diamonds represent the two RX positions, and the blue areas represent the distribution of static obstacles in the five scenarios. The subjects collected an additional 100 movements to study the impact of environmental layout complexity on trajectory reconstruction. Placement 1 represents the original empty room, and Placements 2–6 represent environments with an increasing number of static obstacles. The results, shown in Figure 19b, indicate that the more objects there are in the static environment and the more complex the layout, the greater the error. This is due to the richer multipath environment causing dynamic phase errors in the CSI. Nevertheless, our method can still ensure a high degree of trajectory reconstruction accuracy.

#### 7.2.4. Impact of Transceiver Initial Position

**Figure 20:**
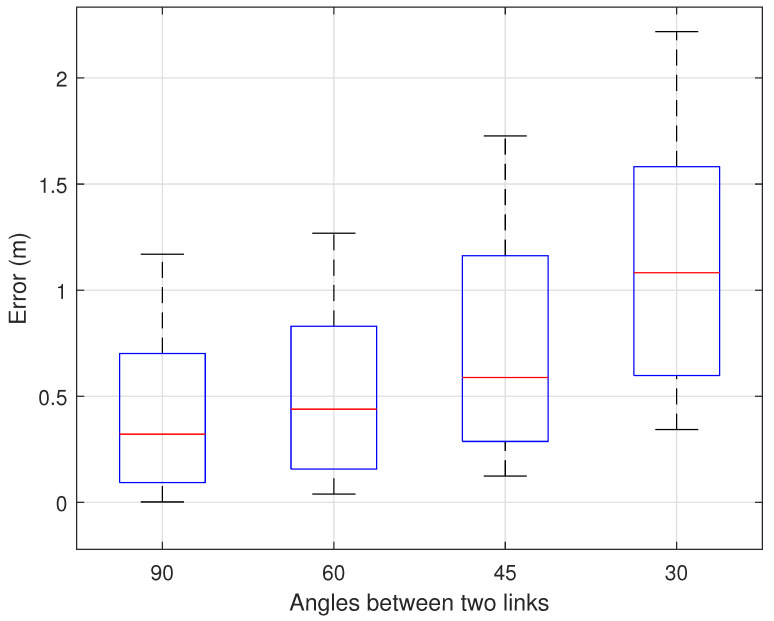
Impact of four different transceiver angles.

Since we determined the movement direction based on the CSI phase changes in the crossing Fresnel zones generated by two non-parallel TX-RX links, the initial positions of the transceivers also affected the experimental results. We additionally set three different initial positions for the transceivers, keeping the LoS distance of the two links constant, with angles of 60 degrees, 45 degrees, and 30 degrees, respectively. Under these conditions, we collected a total of 120 movement trajectory data sets from five test subjects. Figure 20 shows the error results for different initial angles of the transceivers. It can be seen that the smaller the angle between the two transceiver links, the greater the trajectory error. In practical applications, the transceivers should not be set too close to parallel, to prevent a reduction in trajectory reconstruction accuracy.

#### 7.2.5. Impact of User Diversity and Preset Trajectory Diversity

**Figure 21:**
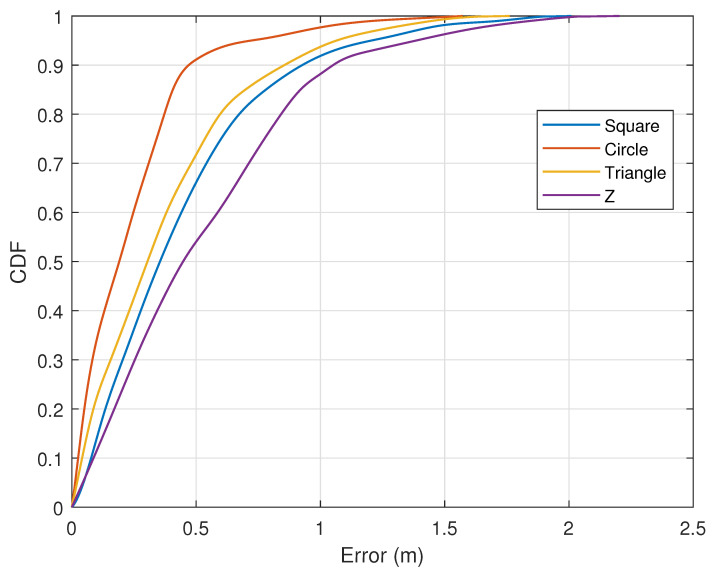
Impact of four different preset trajectories.

**Figure 22:**
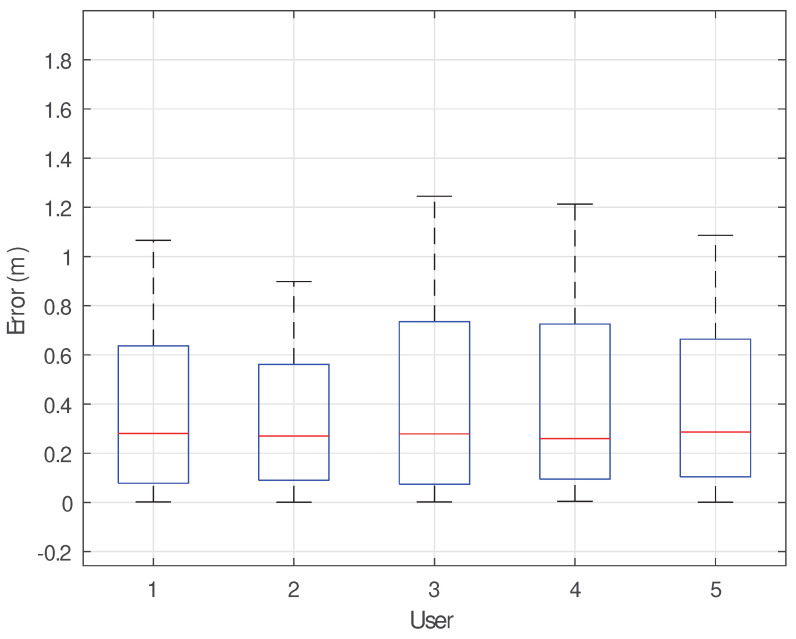
Impact of different users.

We evaluated different preset trajectories of various shapes, including circular, triangular, square, and Z-shaped trajectories. Figure 21 shows that the errors for circular and triangular trajectories are relatively small, while the errors for square and Z-shaped trajectories are slightly larger, making them more prone to deviating from the actual path. This is because circular and triangular trajectories are more likely to simultaneously span the intersecting Fresnel zones, whereas square and Z-shaped trajectories mostly span a smaller Fresnel zone of one link. The accumulated errors in these trajectories are more likely to affect subsequent positioning judgments. Figure 22 shows the trajectory errors of five different test subjects. The results indicate that, even with differences in body size, movement state, gender, etc., our method can still track human movement trajectories with high accuracy. Even for more complex trajectories, the reconstruction error only increases slightly.

#### 7.2.6. Impact of Sample Rate

**Figure 23:**
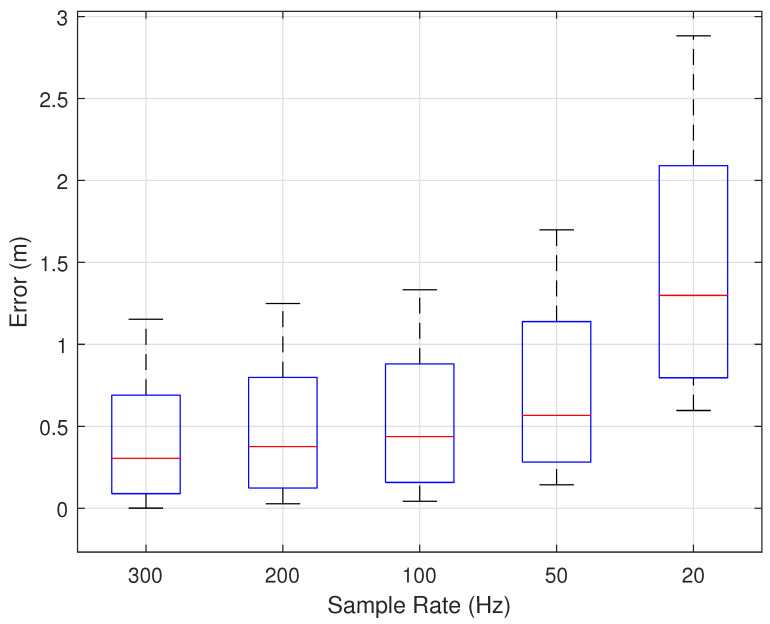
Impact of sample rate.

In our experiments, we used a sampling rate of 300 Hz. However, in practical applications, the sampling rate can be further reduced without significantly affecting accuracy. We evaluated WiPIHT at different sampling rates: 200 Hz, 100 Hz, 50 Hz, and 20 Hz. As shown in Figure 23, the higher the sampling rate, the smaller the trajectory reconstruction error and the higher the accuracy. When the sampling rate is above 50 Hz, the increase in error is not significant and remains within an acceptable range. When the sampling rate is below 50 Hz, the error begins to change significantly. The experiments demonstrate that WiPIHT can accommodate lower sampling rates, significantly reducing computational costs while ensuring real-time trajectory reconstruction within an acceptable error range.

#### 7.2.7. Impact of Other Dynamic Objects

**Figure 24:**
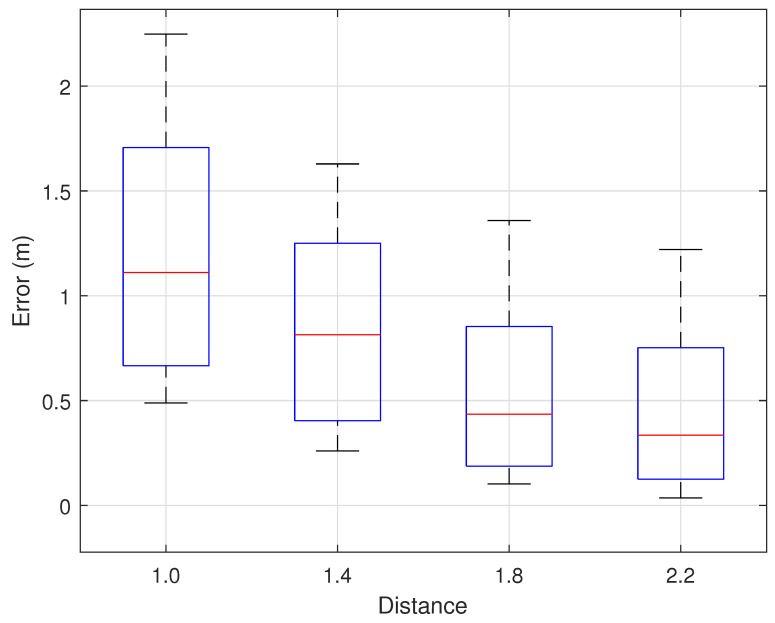
Impact of other dynamic objects.

We evaluated the system’s robustness against other dynamic objects, such as non-test subjects walking around, to verify the system’s reliability. As shown in Figure 15, we arranged interfering personnel at various distances to move around the room to test the impact of dynamic environmental changes on trajectory reconstruction errors. The results are shown in Figure 24. The results indicate that the closer the interfering personnel are to the transceivers, the greater the trajectory reconstruction error. When the interfering personnel are within 1.4 m of the transceivers, the error increases significantly. This also demonstrates that, as long as no other dynamic objects enter the indoor environment, our system maintains strong robustness against interference from personnel engaged in regular activities outside the room.

## 8. Conclusions and Future Work

This paper presents WiPIHT, a position-independent passive indoor human movement trajectory reconstruction system. Using only three commercial WiFi devices and no additional hardware, it enables human tracking. Compared to other WiFi tracking methods, WiPIHT eliminates the dependency on initial positions and transceiver locations. By sensing changes in human movement speed and direction, it derives the relative trajectory shape without requiring extensive data collection and training, significantly reducing costs. Extensive experiments demonstrated that the system achieves high tracking accuracy and can be used for tracking human trajectories in various practical applications.

However, our current method has the following major limitations: Firstly, we estimate the initial human position using the MUSIC algorithm, which relies on the AoA for position determination. Therefore, when a person moves initially along the line connecting the RXs, the same AoA prevents us from calculating the initial position. Secondly, with longer movement trajectories, the accumulated trajectory error increases, gradually affecting subsequent trajectory reconstruction. Additionally, the trajectory shape impacts the error to some extent. Although WiPIHT focuses on 2D planar tracking and validates the core methodology’s effectiveness, its extension to 3D space remains conceptually feasible. However, this expansion faces significant challenges, including the requirement for additional AoA estimation in the vertical dimension, which necessitates either a vertically distributed antenna array or supplementary vertically oriented RXs. Multipath effects become considerably more complex in 3D environments, substantially increasing signal processing complexity. Therefore, developing a viable 3D extension constitutes a significant future research direction for this work. In the future, we will focus on addressing and resolving these issues, making improvements to enable the system to accurately reconstruct target trajectories in three dimensions.

## Figures and Tables

**Table 1:** Comparison of WiFi indoor tracking technologies.

Method	TX-RX Location	Training	Facility	Real Time	Initial Position
RSSI	✓	high	Commercial WiFi	medium	✓
WiDAR	✓	medium	Commercial WiFi	high	✓
IndoTrack	✓	✗	Dedicated device	high	✓
WiPIHT	✗	✗	Commercial WiFi	high	✗

## Data Availability

The raw data supporting the conclusions of this article will be made available by the authors on request.
